# Sound Localization with Hearables in Transparency Mode

**DOI:** 10.3390/audiolres15030048

**Published:** 2025-04-25

**Authors:** Sebastian A. Ausili, Nathan Erthal, Christopher Bennett, Hillary A. Snapp

**Affiliations:** 1 Department of Otolaryngology, University of Miami, 1120 NW 14th Street, 5th Floor, Miami, FL 33136, USA; s.ausili@miami.edu; 2 Department of Music Engineering, University of Miami, 1314 Miller Dr, Coral Gables, FL 33146, USA; nxe244@miami.edu (N.E.); bennett@miami.edu (C.B.)

**Keywords:** binaural hearing, active hearables, AirPods, transparency mode

## Abstract

**Background:** Transparency mode in hearables aims to maintain environmental awareness by transmitting external sounds through built-in microphones and speakers. While technical assessments have documented acoustic alterations in these devices, their impact on spatial hearing abilities under realistic listening conditions remains unclear. This study aimed to evaluate how transparency mode affects sound localization performance with and without background noise. **Methods:** Ten normal-hearing adults completed sound localization tasks across azimuth (±90°) and elevation (±30°) with and without background noise. Performance was assessed with and without AirPods Pro in transparency mode. Sound localization performance was evaluated through linear regression analysis and mean absolute errors. Head-Related Transfer Function measurements quantified changes in binaural and spectral cues. **Results:** While interaural time differences were largely preserved, transparency mode introduced systematic alterations in level differences (up to 8 dB) and eliminated spectral cues above 5 kHz. These modifications resulted in increased localization errors, particularly for elevation perception and in noise. Mean absolute errors increased from 6.81° to 19.6° in azimuth and from 6.79° to 19.4° in elevation without background noise, with further degradation at lower SNRs (*p* < 0.05). Response times were affected by background noise (*p* < 0.001) but not by device use. **Conclusions:** Current transparency mode implementation significantly compromises spatial hearing abilities, particularly in noisy environments typical of everyday listening situations. These findings highlight the need for technological improvements in maintaining natural spatial cues through transparency mode, as current limitations may impact user safety and communication in real-world environments.

## 1. Introduction

The rapid and widespread expansion of hearable devices (e.g., smart wireless electronic devices worn in the ear) represents significant technological advancement in personal audio technology [1,2,3]. Hearables integrate sophisticated functionalities including audio streaming, voice commands, and adaptive noise control, positioning them at the intersection of consumer electronics and assistive hearing technology. With the recent authorization of over-the-counter (OTC) hearing aids by the U.S. Food and Drug Administration (FDA), hearables are uniquely positioned to gain mainstream adoption in the hearing healthcare market, potentially serving millions of individuals with hearing loss [4].

However, the fundamental design of these devices presents significant challenges to natural auditory processing. As it happens with hearing aids and protective hearing devices [5,6,7,8], the physical insertion of hearables into the ear canal modifies its acoustic properties, altering both the resonant frequency and the overall sound pressure at the eardrum [9,10,11]. This modification can significantly alter spatial hearing, which relies on precise acoustic cues for accurate sound source localization. These cues include interaural time differences (ITDs) predominant at lower frequencies (<1.5 kHz), interaural level differences (ILDs) at higher frequencies (>3 kHz), and spectral cues above 4 kHz that emerge from complex acoustic interactions with the torso, head, and pinnae [12].

Conventionally, headphones lacked active electronics for real-time audio processing and functioned as passive devices. By sitting in or on the ear, and similar to the effects of passive earplugs, they altered binaural acoustic interactions and disrupted pinna-related spectral cues impacting sound localization [5,13,14]. To address these acoustic modifications, manufacturers have implemented “transparency” or “hear-through” modes that utilize built-in microphones to capture and process ambient sounds in real-time. The theoretical objective of these systems is to achieve acoustic transparency, where the transfer function measured at the eardrum should remain consistent between the unoccluded condition and with the device inserted. This approach has been adopted in both traditional hearing aids and modern consumer hearables to minimize the acoustic disruptions caused by ear canal occlusion, with the goal of preserving natural spatial hearing and maintaining a sound experience similar to that of an open ear [10,14,15,16,17,18,19].

Recent acoustic measurements of commercial hearables’ transparency features have revealed substantial deviations from natural sound transmission characteristics, showing significant alterations in both monaural and binaural cues [20,21]. The deviations from natural hearing included processing delays ranging from 0 to 9.7 ms, with some devices showing left-right channel asymmetries up to 9.5 ms, and frequency response deviations of up to 15 dB relative to the open-ear configuration. While some devices maintained better acoustic transparency than others, significant compromises in frequency response accuracy and spatial cue preservation were evident in all studies, highlighting the technical challenges in achieving natural sound transmission through hearables. While these acoustic measurements provide crucial insights into signal modification, complementary perceptual studies have examined user experience through sound quality ratings assessments, demonstrating variable outcomes across different device implementations and listening scenarios [22]. However, despite extensive objective measurements, the extent to which these acoustic alterations translate to real-world localization deficits, particularly in the presence of environmental noise, remains unclear. Recent work by Watanabe and Terada (2023) [11] provides important behavioral evidence on spatial hearing degradation when using transparency mode for some of the available commercial hearables, demonstrating a significant decline in horizontal sound localization accuracy from 91.5% (open ear) to 68.9% (device with transparency mode). Although this work provided important information on transparency mode’s impact on spatial hearing, its evaluation was limited to horizontal-plane localization under ideal acoustic conditions. Critical aspects of real-world listening such as elevation perception and the effects of background noise have yet to be explored.

The presence of background noise introduces additional challenges to spatial hearing. Processing delays and potential hearables’ misalignment between ears can affect ITD cues, while environmental noise may interact with the devices’ output through acoustic leakage, potentially resulting in comb-filtering effects [20]. Despite these real-world challenges, current evaluations of hearables’ transparency modes have predominantly occurred under idealized acoustic conditions, limiting our understanding of their performance in everyday listening scenarios.

The present study addresses this critical gap by conducting a comprehensive evaluation of how active hearables affect spatial hearing abilities with and without background noise. We employ a dual-methodology approach that uniquely combines objective head-related impulse response (HRIR) measurements with behavioral assessments of localization ability in normal-hearing listeners. By examining performance across different signal-to-noise ratios (SNRs), we quantify how these devices maintain spatial cues under conditions that challenge the auditory system’s natural localization mechanisms. This integrated acoustic and behavioral investigation provides insight into the effectiveness of current transparency mode implementations and their implications for maintaining spatial awareness in noisy environments.

## 2. Methods

### 2.1. Listeners

Participants included 10 adults ranging in age from 22 to 35 years (median = 24; 3 female). All participants had normal hearing as confirmed by a standard pure-tone audiogram [23], where their hearing was within 20 dB of audiometric zero across the independent standard audiometric test frequencies, 250–8000 Hz, and no prior history of hearing impairment. The experimental procedures were approved by the Institutional Review Board of the University of Miami (20211149), and all participants provided informed consent.

### 2.2. Hearable Device

Participants were fitted with first-generation Apple AirPods Pro (Model A2084, Apple Inc., Cupertino, CA, USA) for the experimental protocol. The transparency mode was set, which permits environmental sound passthrough while maintaining a balanced acoustic profile. At the time of data collection, devices were running the manufacturer’s most recent firmware version, ensuring standardized operational parameters. The transparency mode has a minimal audio processing latency to maintain real-time acoustic perception. No individual device-specific modifications were implemented that could introduce variability in the acoustic signal processing, thus maintaining consistent experimental conditions.

Proper insertion was verified using the built-in ‘Ear Tip Fit Test’ in the AirPods settings. The process involves playing a specific music track through the earbuds while built-in microphones measure the acoustic response within the ear canal to assess seal quality and leakage. If the fit was not ideal, the AirPods were re-positioned until they passed the check.

### 2.3. Setup

The experiments took place in a sound-attenuated auditory booth (4.3 × 4.3 × 2 m) equipped with an array of 72 speakers (e301, KEF, Maidstone, UK) arranged in a 1.3 m radius. Target sounds were presented from 47 speakers spanning −90° to 90° in azimuth and −30° to 30° in elevation. Audio routing was accomplished through a Focusrite RedNet PCIeR (Focusrite, High Wycombe, UK) connected to a set of 3 Sonible d:24 multi-channel amplifiers (Sonible, Graz, Austria) with a sampling rate of 44.1 kHz. Stimuli presentation and the analysis of the responses were implemented in MATLAB (ver. R2022a, The MathWorks, Natick, MA, USA) using the Psychtoolbox-3.0.19 extension [24,25,26]. Lab Streaming Layer library (https://github.com/sccn/labstreaminglayer; accessed on 10 March 2020) was implemented for device time synchronization. All levels were measured using a sound pressure level meter (NSRTW mk4, Convergence Instrument, Sherbrooke, QC, Canada) positioned at the center of the speaker array.

### 2.4. Objective HRIR Measures

To analyze the impact of the hearables on the spatial cues (i.e., ILDs, ITDs, and spectral cues), HRIRs were recorded in silence and noise, with and without the AirPods.

#### Stimuli and Procedures

Binaural recordings were obtained by positioning the dummy head (Neuman KU 100, Georg Neumann GmbH, Berlin, Germany) at the center of the speaker array. The binaural dummy head has physical approximate dimensions of 280 mm × 180 mm × 220 mm, and includes two microphone capsules built into its anatomically realistic pinnae (20 Hz to kHz). Data were registered through a Focusrite RedNet MP8R (Focusrite, High Wycombe, UK) at a sample rate of 44.1 kHz. Proper positioning was verified by ensuring equidistance from speakers at −90° and 90°, and equivalent distance from both ears to speakers at 0° and 180°. Logarithmic sine sweeps (15 s, 20 Hz to 20 kHz) were presented at 70 dBA from ±90° in steps of 10°. Additional recordings were made at 50, 60 and 70 dBA with constant Gaussian white noise (55 dBA) to achieve signal-to-noise ratios of −5 dB, 5 dB, and +15 dB. Binaural HRIRs were then obtained by convolving the recorded sweeps with the inverse of the original sweep [27]. All measurements were repeated with Apple AirPods Pro inserted into the dummy head’s ears.

ITDs were extracted through a multi-stage filtering process. First, a linear phase finite impulse response (FIR) bandpass filter (0.1–1.5 kHz) was applied to the HRIRs to preserve phase information in the frequency range most relevant for ITD processing. The filtered signals underwent half-wave rectification, followed by a 1.4 kHz Brickwall lowpass filter to remove high-frequency components [28]. ITDs were then computed by identifying the peak of the cross-correlation function between the left and right filtered HRIRs from corresponding sweeps [29]. The cross-correlation was implemented using MATLAB’s xcorr function with a maximum lag parameter of ±1 ms, chosen to encompass the physiological limit of human ITDs (~800 µs) [30].

ILDs were extracted by applying an infinite impulse response (IIR) bandpass filter (3–20 kHz) to the HRIRs, focusing on the frequency range where head-shadow effects are most prominent. The ILD values were then computed as the difference in root mean square (RMS) amplitude between the filtered left and right HRIRs.

Lastly, monaural spectral cues were characterized by computing the magnitude responses of the HRIRs for each elevation angle (ranging ±30° in steps of 15°) using Fast Fourier Transform (FFT) analysis between 3 kHz and 16 kHz, encompassing the frequency range critical for elevation perception.

### 2.5. Behavioral Localization Measures

To examine how transparency mode in hearables affects spatial hearing abilities, we conducted behavioral sound localization experiments in normal-hearing listeners under various acoustic conditions. We examined both azimuthal and elevation localization abilities across different signal types and noise conditions.

#### 2.5.1. Procedures

**Figure 1:**
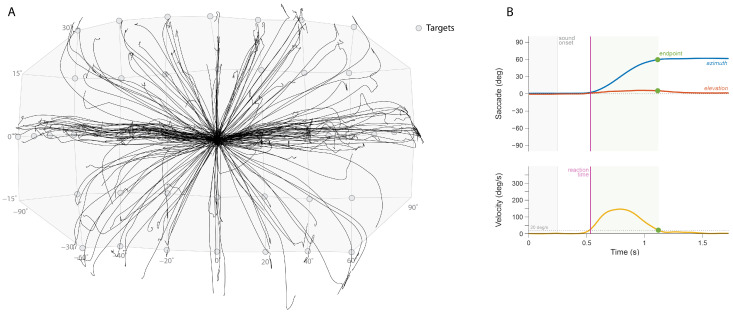
(**A**) Head trajectories for each trial (represented by line), localizing stimuli generated from the sound sources (represented by circles). (**B**) Example measurement of a subject’s head orientation and velocity for one trial.

Subjects sat comfortably in a chair positioned at the center of the speaker array, facing 0° azimuth and 0° elevation. The room was kept completely dark to eliminate visual cues and prevent response bias from known speaker locations. Head orientation was tracked using a camera system consisting of 6 trackers (Flex 3 Optitrack, Natural Point, Corvallis, OR, USA) and a custom headband with reflective markers (see Figure 1A,B). Additionally, the headband was equipped with a laser pointer to help participants align their head and eye gaze with a central LED light mounted on the reference loudspeaker.

For each trial, participants first aligned their laser pointer with the central LED and pressed a button to indicate readiness. Upon button press, the LED turned off and after a randomized delay (200–300 ms), the stimulus was presented from a random speaker location within the front hemifield (spanning ±90° azimuth and ±30° elevation). Participants were instructed to point their head toward the perceived sound source location as quickly and accurately as possible. Once the head orientation was recorded, the central LED reactivated, signaling participants to return to the starting position for the next trial.

#### 2.5.2. Test Conditions

Two primary experimental conditions were tested: normal hearing (NH) and with AirPods Pro in transparency mode. Each condition was evaluated both in silence and with background noise. In silent conditions, the target stimulus consisted of a 150 ms Gaussian white noise burst filtered into three frequency ranges: broadband (BB, 0.2–20 kHz), low-pass (LP, 0.2–1.5 kHz), and high-pass (HP, 3–20 kHz). While LP and HP filtered noise bursts were presented at a fixed level of 60 dBA, the BB noise was tested at three intensities: 50, 60, and 70 dBA.

For conditions with background noise, the target stimulus was a 150 ms broadband buzzer noise presented at three intensities (50, 60, and 70 dBA) against continuous Gaussian white noise fixed at 55 dBA. This configuration produced three signal-to-noise ratios (SNRs): −5, +5, and +15 dB. Background noise was spatially distributed through eight speakers positioned at ±30°, ±70°, ±110°, and ±150° in azimuth (0° elevation) surrounding the listener. Target stimuli were presented following a randomized delay of 200–300 ms after noise onset.

#### 2.5.3. Data Analysis

Head orientation trajectories were recorded throughout each trial (Figure 1A). Movement onset (defining reaction time) and offset (defining final response position) were determined using a velocity threshold criterion of 20 degrees/s (Figure 1B). Sound localization performance was quantified through linear regression analysis using a least-squares error criterion. For both azimuth and elevation dimensions, the relationship between target azimuth/elevation angles (*α*_T_ and *ε*_T_, respectively) and response azimuth/elevation angle (*α*_R_ and *ε*_R_) was obtained using the following equations:

(1) $$\alpha_{R}=b_{\alpha }+g_{\alpha }\times \alpha_{T}\;e_{R}=b_{e}+g_{e}\times e_{T}\;$$

Here, g represents the slope or gain of the best-fit regression line (dimensionless) and b the intercept or bias (in degrees). We also computed the coefficient of determination (r^2^), or goodness of fit, for all linear fits. Note that a perfect localization response should yield a gain of 1, bias of 0° and a high coefficient of determination of 1.0.

Furthermore, to quantify an overall measure of the response accuracy, we also used the mean absolute error (MAE) across trials, according to the following equations:

(2) $${MAE}_{\alpha }=\frac{1}{N}\sum_{n=1}^{N}\left|\alpha_{R}^{n}-\alpha_{T}^{n}\right|\;{MAE}_{e}=\frac{1}{N}\sum_{n=1}^{N}\left|e_{R}^{n}-e_{T}^{n}\right|$$

with *N* representing the total number of trials and *n* representing each individual trial.

Reaction times (RTs) were calculated as the difference between the onset of the auditory target and the onset of the participant’s head movement (measured in milliseconds). As is typical in human response data, RTs followed a positively skewed distribution due to a subset of longer-latency trials. To normalize this distribution and allow for more robust statistical analyses, we transformed reaction times into their reciprocals (1/RT, in s^−1^), a measure known as promptness [31,32,33]. This transformation yields a distribution that more closely approximates normality, where higher promptness values reflect faster responses, and lower values reflect slower reactions.

### 2.6. Statistical Analysis

We analyzed the data using estimation statistics to provide a comprehensive understanding of effect sizes and their precision [34]. Specifically, we employed a bias-corrected and accelerated bootstrap resampling technique to estimate the 95% confidence intervals of the mean differences. The analysis was conducted using the DABEST (Data Analysis with Bootstrap-Coupled Estimation) package in MATLAB [34]. Throughout the Results section, data distributions are presented using violin plots, which illustrate the full range of the data along with the 25th and 75th percentiles and the median. Individual data points were plotted for each group and tested condition.

To assess statistical significance, we performed permutation t-tests with 5000 reshuffles of control and test labels for each *p*-value calculation. Due to the non-normality of the data, a permutation approach is used to provide a more robust analysis for the skewed distributions. This method presents a clearer understanding of the interventions’ effect size and its precision, thereby providing a more reliable inference framework [34]. The reported *p*-values represent the probability of observing the observed effect sizes under the null hypothesis of zero difference.

## 3. Results

### 3.1. Objective HRIR Measures in Quiet

**Figure 2:**
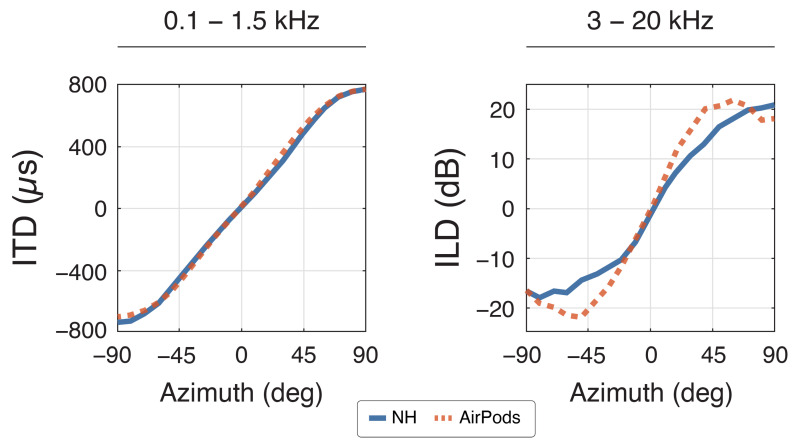
Binaural cues compared between NH (blue) and AirPods (red) with ITDs (left) and ILDs (right) along the azimuth plane at 0° elevation. Positive ITDs mean sound reaching the right side first, and vice versa. Similarly, positive ILDs mean a louder signal on the right, and vice versa.

Analysis of the binaural cues derived from measured HRIRs revealed distinct patterns across device conditions in the horizontal plane (±90°; Figure 2). Under NH condition, ITDs showed the expected monotonic variation with azimuth, spanning the physiologically relevant range of ±700 µs. Due to the acoustic leakage in the low frequencies, the use of AirPods does not alter temporal relationships, demonstrating deviations from NH condition of less than 45 µs across all measured angles. In contrast, ILDs showed substantial alterations when measured through the AirPods compared to NH. The most pronounced deviations manifested as systematic amplification of the natural level differences, with maximum increases of 8.0 dB and 7.5 dB observed at −50° and 40° azimuth, respectively. This pattern of ILD enhancement diminished near the median plane, where differences between conditions were constrained to 0.5–2.0 dB, suggesting a spatial dependence in the device’s impact on interaural intensity cues.

**Figure 3:**
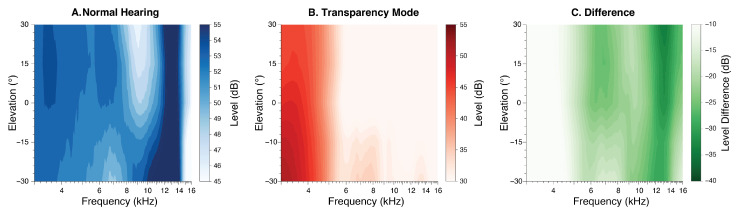
(**A**) Monaural spectral pinna cues for NH and (**B**) AirPods for −30° to 30° elevation and (**C**) their difference.

The monaural spectral cues were substantially altered when comparing NH to the AirPods condition (Figure 3). As expected, the characteristic spectral notches and peaks above 5 kHz were eliminated by the AirPods.

### 3.2. Sound Localization in Quiet

**Figure 4:**
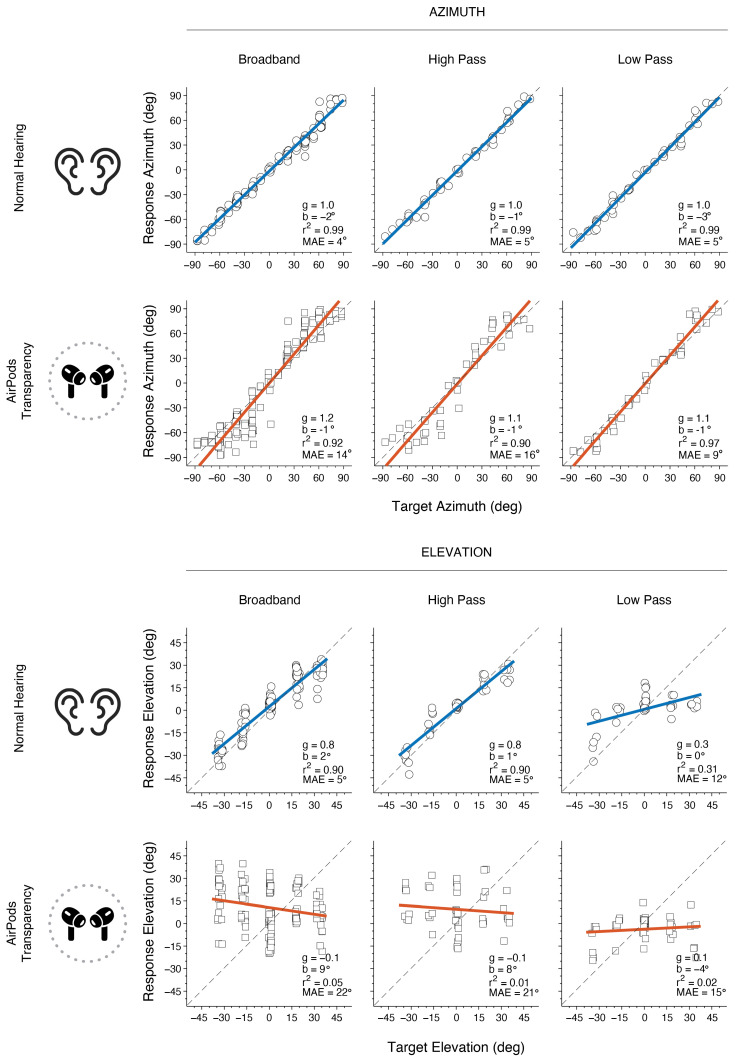
Example of Subject 9 results for azimuth and elevation localization for different stimuli in each listening condition. Circles and squares represent the individual responses for azimuth and elevation, respectively. The colored line shows the linear regression (NH in blue, AirPods in red), and the dashed line represents a perfect unity line. Each plot displays the gain of the best-fit regression line (g), the bias (b), the correlation coefficient (r^2^), and the mean absolute error (MAE).

Sound localization performance was evaluated in quiet for BB (50, 60, and 70 dBA), LP (60 dBA), and HP (60 dBA) (see Methods). Figure 4 depicts an example of a participant’s sound localization results in each hearing condition. The listener accurately localizes horizontal sound sources in the NH condition, achieving g_α_ values of 1, b_α_ close to 0, r^2^_α_ of ~1 and MAE_α_ ≤ 5° across all stimuli. In the AirPods listening condition, azimuth responses are mostly accurate for the LP stimuli, yielding a bigger spread on the responses for BB and HP stimuli. In general, azimuth MAE with AirPods showed a bigger overall MAE than in the NH condition (≥9°), being the highest for BB and HP (14° and 16°, respectively). In elevation, the listener showed an expected normal-hearing outcome with target–response relationships for BB and HP, and with poor localization in LP due to lack of monaural spectral pinna cues in that frequency range. However, when examining the elevation responses with the AirPods, poor performance is observed across all stimuli. In this case, gains were practically 0, demonstrating the absence of a target–response relationship, together with large MAEs and low correlation values (i.e., big data spread).

**Figure 5:**
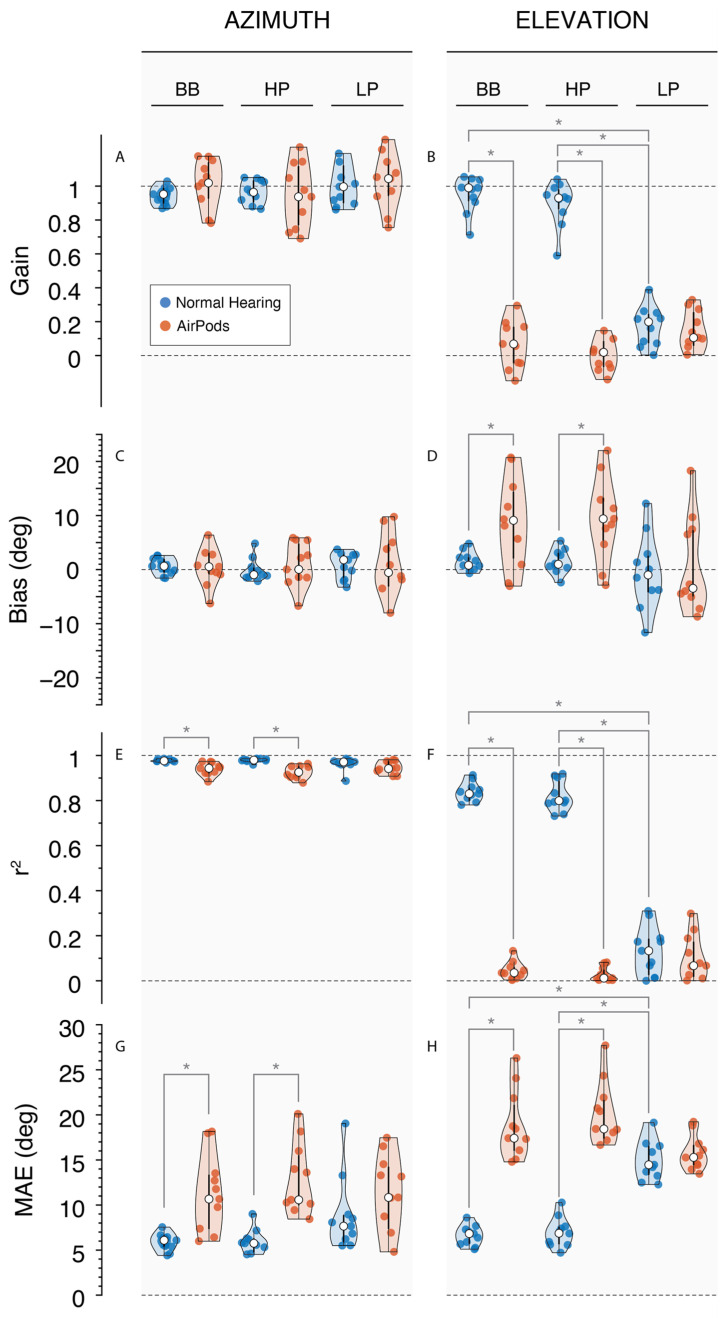
Violin plots presenting each participant’s performance (colored circles) for each stimulus, with the 25th and 75th percentiles indicated by the black bars and the median indicated by the white circle. Responses under NH conditions (blue) and AirPods (red). Left column: responses in azimuth for (**A**) Gain, (**C**) Bias, (**E**) r^2^, and (**G**) MAE; right column: responses in elevation for (**B**) Gain, (**D**) Bias, (**F**) r^2^, and (**H**) MAE. * indicates *p*-value < 0.05.

Overall results for sound localization performance without background noise for all listeners are shown in Figure 5. In the NH condition, listeners generally demonstrated accurate sound localization in azimuth and elevation in the NH condition. Across all stimuli, azimuth gain was approximately 1 (g_α_ = 0.97 ± 0.31), bias was close to 0° (b_α_ = 0.72° ± 1.52°), correlation was high (r^2^_α_ = 0.97 ± 0.01) and overall error was low (MAE_α_ = 6.81° ± 0.99°). Sound localization in the vertical plane also yielded expected results, as BB and HP yielded high gains (g_ε_ = 0.93 ± 0.05), bias close to 0 (b_ε_ = 1.42° ± 0.96), reasonable correlation (r^2^_ε_ = 0.83 ± 0.03) and low error (MAE_ε_ = 6.79° ± 0.59°). Low-frequency vertical sound localization was poor as expected, evidenced by a low regression slope (g_ε_ = 0.17 ± 0.08), low correlation (r^2^_ε_ = 0.13 ± 0.07) and high error (MAE_ε_ = 15° ± 1.43°). Data showed no significant differences across BB levels on either condition for azimuth: g_α_ (*p* > 0.06), bα (*p* > 0.08), r^2^_α_ (*p* > 0.06), or MAE_α_ (*p* > 0.09). Similarly, there were no significant differences across BB levels for elevation values: g_ε_ (*p* > 0.26), bε (*p* > 0.33), r^2^_ε_ (*p* > 0.14), MAE_ε_ (*p* > 0.11). Therefore, BB results were pooled across levels for azimuth and elevation.

When listening through AirPods, data generally showed a larger spread and higher localization errors than NH. This resulted in significant differences between AirPods and NH r^2^_α_ for BB (*p* < 0.001) and HP (*p* < 0.001), but not for LP (*p* = 0.103). Moreover, MAEs for the AirPod conditions were higher than NH for BB (*p* < 0.05) and HP (*p* < 0.001) but, again, did not reach significance for LP (*p* = 0.053). A review of the data demonstrates large overall errors of two subjects in the NH condition, which may explain the non-marginal statistical difference for the LP stimuli (see Figure 5G, LP).

Noticeably, vertical sound localization gain with the AirPods was poor across stimuli (*g_ε_* = 0.07 ± 0.05) and significantly different than NH for BB and HP (*p* < 0.001). This condition also elicited a significant perceptual change upward in bias for BB and HP (*b_ε_* = 9.01° ± 3.41°; *p* < 0.05). Similarly, due to the spread of the data, there was a substantial drop in BB and HP correlation values (*r*^2^*_ε_* = 0.04 ± 0.02; *p* < 0.001). Lastly, the same frequencies exhibited a significant increase in error (MAE*_ε_* = 19.4° ± 1.59°; *p* < 0.001), reaching more than double their error in the NH condition.

### 3.3. Objective HRIR Measures in Background Noise

**Figure 6:**
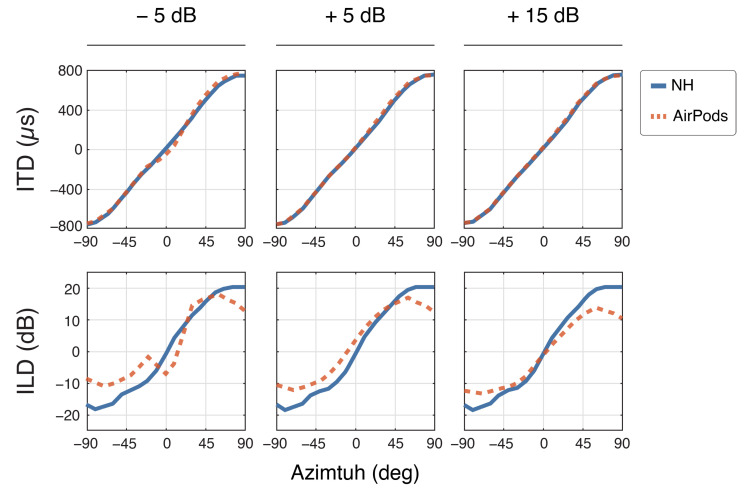
Binaural cues for normal hearing (NH) and AirPods across all SNR levels. The top row shows interaural time differences (ITDs) for frequencies between 0.1 kHz and 1.5 kHz, while the bottom row displays interaural level differences (ILDs) for frequencies from 3 kHz to 20 kHz.

Figure 6 presents the binaural cues measurements obtained for each SNR level in background noise (see methods). ITDs and ILDs remained consistent throughout each SNR level for the NH recordings. With the AirPods, ITDs remain mostly preserved despite decreasing SNR, with the largest deviation of −113 µs at 0° for the ITD at −5 SNR. However, ILDs exhibited a reduced range, with values between −11 dB and 14 dB, compared to the NH range of −17 dB to 20 dB. As SNR decreased, there was a noticeable bias approaching +90° at +5 dB (+4 dB at 0° azimuth), and at −5 dB SNR, errors shifted toward the medial plane. Additionally, at −5 dB SNR, the characteristic sigmoid curve was disrupted between 0° and ±10° azimuth.

### 3.4. Sound Localization in Background Noise

**Figure 7:**
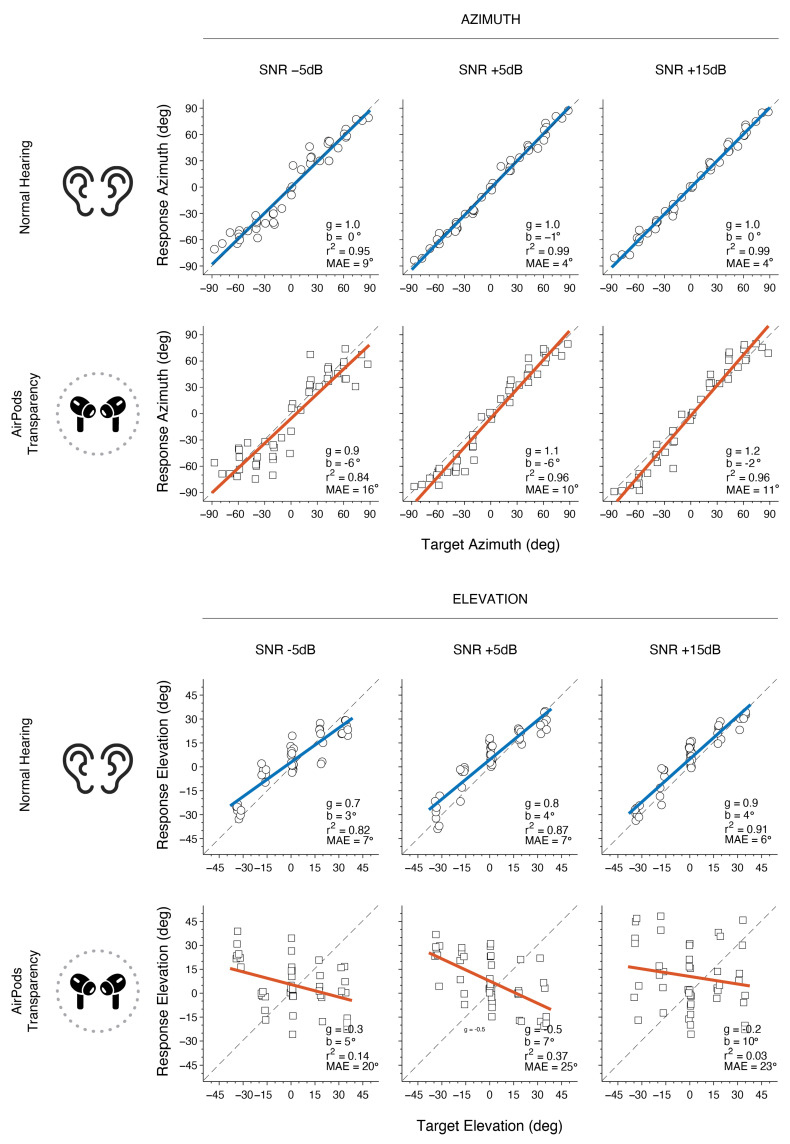
Example results for Subject 9 showing azimuth and elevation localization across different SNR levels for each listening condition. Circles represent azimuth responses, while squares denote elevation responses. Colored lines indicate linear regression fits (blue for NH, red for AirPods), with the dashed line representing perfect unity.

In Figure 7, the same participant as presented in Figure 4 is shown under different SNR conditions. For NH, the results indicate accurate azimuth localization for +5 dB and +15 dB SNR levels, while minor performance decrease is observed at −5 dB SNR, indicated by a decrease in r^2^_α_ and an increase in MAE_α_. When using AirPods, MAE_α_ increased by at least 6° overall when compared to the NH condition. Similar to NH, azimuth localization with AirPods was generally less accurate at lower SNRs, with a larger decrease of 0.12 in r^2^α and an increase of 6° in MAE_α_ from +5 dB to −5 dB SNR. As expected, vertical localization in noise for NH was consistently lower in accuracy than for azimuth. Performance was relatively close to target values at +15 dB SNR but gradually declined as SNR decreased. With AirPods, localization accuracy in elevation was poor across all SNRs (−5, +5, and +15 dB), showing consistently low gain values, indicating a lack of a reliable target-response relationship. These results yield large MAE_ε_ and low r^2^ε values, demonstrating substantial variability and error in elevation localization.

**Figure 8:**
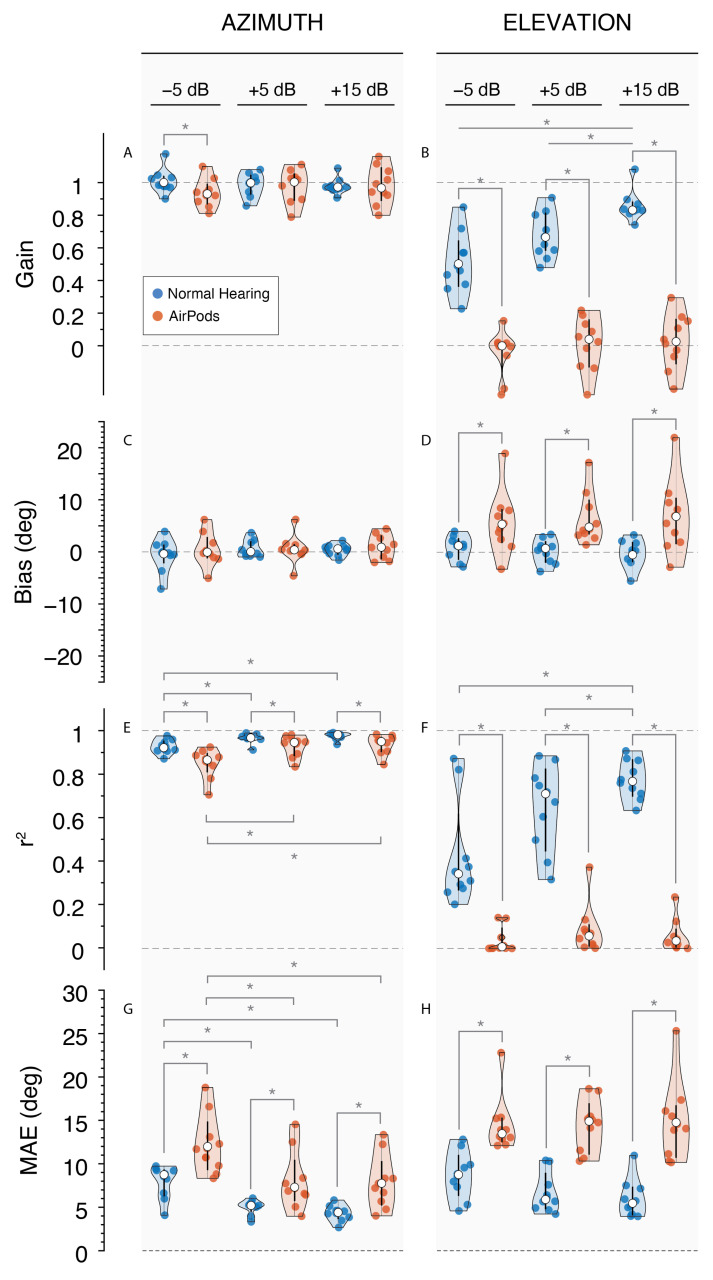
Violin plots of sound localization outcomes in noise for NH and AirPod listening conditions at each SNR level. Left column: responses in azimuth for (**A**) Gain, (**C**) Bias, (**E**) r2, and (**G**) MAE; right column: responses in elevation for (**B**) Gain, (**D**) Bias, (**F**) r2, and (**H**) MAE. * indicates *p*-value < 0.05.

Overall results for sound localization performance in background noise are shown in Figure 8. In the NH condition, listeners generally demonstrated accurate azimuth localization across all SNRs, with near-perfect gain (g_α_ = 0.99 ± 0.06), minimal bias (b_α_ = 0.13° ± 2.11°), high correlation (r^2^_α_ = 0.96 ± 0.02), and low error (MAE_α_ = 7.58° ± 1.45°). However, as SNR decreased, a decline in performance was observed in both r^2^_α_ and MAE_α_ (*p* < 0.05). This SNR change appeared to have a larger impact on vertical sound localization. Besides elevation bias (*p* = 0.628), gain (g_ε_; *p* < 0.001), goodness of fit (r^2^_ε_; *p* < 0.001), and mean absolute error (MAE_ε_; *p* < 0.05) showed significantly poorer outcomes as SNR decreased.

Localization in noise with AirPods yielded an overall poorer performance compared to NH. Although azimuth gain was only significantly different at −5 dB SNR (*p* < 0.001), MAE_α_ and r^2^_α_ were poorer in all SNRs (*p* < 0.001). This was not the case for azimuth bias between listening conditions (*p* > 0.387). As SNR decreased, increased response variability and errors were reflected in r^2^_α_ and MAE_α_. The azimuth goodness of fit (r^2^_α_) was 0.93 ± 0.02 at +15 dB SNR, but significantly dropped to 0.84 ± 0.03 at the lowest SNR (*p* < 0.05). Similarly, the MAE_α_ increased from 8.2° ± 1.1° at +15 dB SNR to 19.6° ± 4.6°, showing a significant inverse relationship with SNR.

As expected, elevation localization with AirPods was significantly worse in noisy conditions than NH. Significant differences were observed across all SNR levels, including reduced gain (g_ε_ = −0.02 ± 0.17; *p* < 0.001), increased bias (b_ε_ = 7.9° ± 6.5°; *p* < 0.05), decreased correlation (r^2^_ε_ = 0.07 ± 0.07; *p* < 0.001), and higher mean absolute error (MAE_ε_ = 19.6° ± 4.4°; *p* < 0.001). In this condition, SNR did not affect g_ε_ (*p* = 0.315), b_ε_ (*p* = 0.714), r^2^_ε_ (*p* = 0.386), or MAE_ε_ (*p* = 0.787).

### 3.5. Sound Localization Promptness

**Figure 9:**
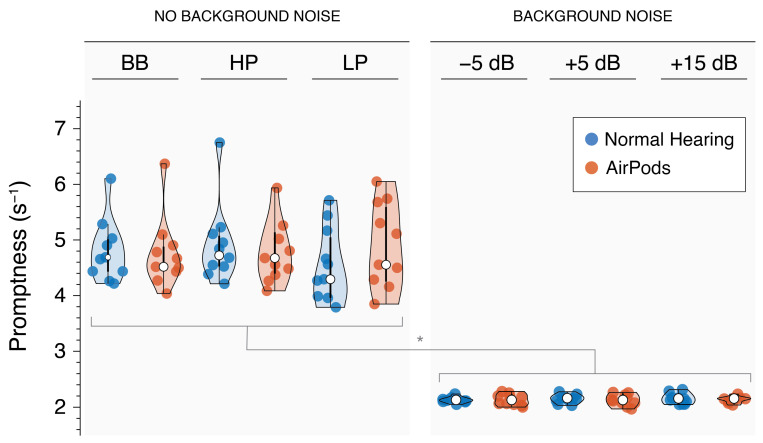
Violin plots showing response promptness for NH (red) and AirPods (blue) listening conditions during sound localization, both with and without background noise. * indicates *p*-value < 0.05.

Response promptness by group and for sound localization with and without noise is shown in Figure 9. Listeners displayed increased promptness on the localization tasks performed in quiet (M = 4.77 ± 0.15 s^−1^) compared to in background noise (M= 3.64 ± 0.06 s^−1^; *p* < 0.001). Specifically, listeners are faster to initiate the localization response where there is no competing noise. Review of Figure 9 reveals greater variance in the response times of listeners in quiet compared to in noise, likely reflecting the difference in cognitive demand allowing for more fluctuations and individualized differences in response patterns, whereas competing noise requires more focus narrowing the window of response times. There was no significant effect of stimulus type for localization in quiet conditions (Figure 9, left panel; *p* > 0.05) or by SNR (Figure 9, right panel; *p* > 0.05).

## 4. Discussion

Spatial hearing is essential for effective interaction with our environment, as it allows individuals to localize sound sources, navigate safely, and respond appropriately to auditory information. Our study provides a comprehensive assessment of how transparency mode in commercial hearables affects spatial hearing abilities under both ideal and challenging acoustic conditions—a critical gap identified in previous research. Through combined objective and behavioral measures, we demonstrate significant alterations in spatial cues and their perceptual consequences.

As active hearables grow in popularity and technical sophistication, they are increasingly replacing passive headphones that lack spatial awareness capabilities. The development of transparency mode represents a key milestone in this evolution, aimed at restoring acoustic cues critical for environmental perception and user safety. However, consistent with prior technical measurements, our study found that spatial cues were compromised when using AirPods in transparency mode [11,20,21]. The preservation of ITDs can be attributed to acoustic leakage in low frequencies, allowing direct acoustic signals to reach the ear canal. In contrast, ILDs showed systematic deviations from the open ear pattern, particularly in the presence of background noise. These changes were likely influenced by the additional processing required for the real-time pass-through audio algorithm and the effect of background noise on this processing. As expected, monaural pinna cues for elevation were completely disrupted above 5 kHz due to the unavoidable alterations in acoustic interactions between sound waves and the natural shape of the pinnae caused by the hearables.

Subjects’ sound localization performance reflected the impact of compromised auditory spatial cues. Vertical sound localization was completely disrupted across all listening conditions with AirPods. The absence of vertical cues is crucial not only for up–down localization but also for front–back sound discrimination. The safety concerns associated with using hearable devices remain even with the transparency mode, as detecting sounds from behind relies solely on auditory input without visual support. Notably, these devices are primarily used by individuals without hearing loss who typically have no difficulty perceiving sounds from the front or back. Using a hearable in transparency mode can impair this capacity, effectively creating a limitation that would not otherwise exist.

Azimuth localization was significantly affected for sound stimuli with a high-frequency range (>3 kHz), with some responses showing errors exceeding 40°. These errors were further exacerbated by background noise, particularly at negative SNRs, highlighting potential challenges in real-world environments. Interestingly, response promptness was significantly reduced in the presence of background noise but remained unaffected while listening through hearables (Figure 9). This finding aligns with previous research showing that noise can degrade performance and increase reaction times during localization tasks [35]. In contrast, the acoustic modifications introduced by the hearables’ transparency mode did not appear to hinder response speed. This suggests that, for NH listeners, these devices do not substantially increase listening effort under the tested conditions. As reaction time can reflect underlying cognitive demand and listening effort [36,37], these results suggest that background noise imposes a measurable increase in effort, whereas using AirPods, regardless of the condition, might not alter the cognitive load required for spatial hearing.

The implications of our findings extend beyond consumer applications to the emerging role of hearables as over-the-counter hearing aids. Interestingly, sound localization performance with AirPods in transparency mode is comparable to the levels reported for hearing aid users in the literature [38,39,40]. As active hearables become more accessible and affordable compared to traditional hearing aids, these initial comparable outcomes are promising. However, for a more comprehensive evaluation, listeners with different types of hearing loss should be tested to validate these findings.

Our integrated acoustic and behavioral approach reveals that while transparency mode enables environmental awareness, it introduces significant compromises in spatial hearing abilities that may impact user safety and listening experience. These findings highlight the need for technological improvements in maintaining natural spatial hearing cues through transparency mode, particularly for high-frequency spectral cues critical for elevation perception. Despite these current limitations, mass-produced hearable technology may offer some of the same benefits as more expensive devices for individuals with hearing impairment, potentially improving accessibility to hearing healthcare. Future research should evaluate spatial hearing performance in listeners with hearing loss, focusing not only on sound localization but also on spatial speech understanding in noise—a critical aspect of real-world listening that will help validate these devices’ effectiveness across diverse user populations and listening environments.

## Data Availability

Data can be made available upon request, subject to the discretion of the authors.

## References

[1] Plazak J., Kersten-Oertel M. (2018). A Survey on the Affordances of “Hearables”. Inventions.

[2] Crum P. (2019). Hearables: Here Come the: Technology Tucked inside Your Ears Will Augment Your Daily Life. IEEE Spectr..

[3] IDC Wearable Devices Market Insights. https://www.idc.com/promo/wearablevendor.

[4] FDA FDA Authorizes First Over-the-Counter Hearing Aid Software. https://www.fda.gov/news-events/press-announcements/fda-authorizes-first-over-counter-hearing-aid-software.

[5] Kayser H., Ewert S.D., Anemüller J., Rohdenburg T., Hohmann V., Kollmeier B. (2009). Database of Multichannel In-Ear and Behind-the-Ear Head-Related and Binaural Room Impulse Responses. EURASIP J. Adv. Signal Process..

[6] Durin V., Carlile S., Guillon P., Best V., Kalluri S. (2014). Acoustic Analysis of the Directional Information Captured by Five Different Hearing Aid Styles. J. Acoust. Soc. Am..

[7] Zimpfer V., Sarafian D. (2014). Impact of Hearing Protection Devices on Sound Localization Performance. Front. Neurosci..

[8] Kroener L., Garcia A., Zimpfer V., Langrenne C. Hearing Protections: Effects on HRTFs and Localization Accuracy. Proceedings of the 23rd International Congress on Acoustics.

[9] Denk F., Kollmeier B. (2021). The Hearpiece Database of Individual Transfer Functions of an In-the-Ear Earpiece for Hearing Device Research. Acta Acust..

[10] Liebich S., Vary P. (2022). Occlusion Effect Cancellation in Headphones and Hearing Devices—The Sister of Active Noise Cancellation. IEEE ACM Trans. Audio Speech Lang. Process..

[11] Watanabe H., Terada T. (2023). Transparency Mode of Hearable Reduces Your Spatial Hearing: Evaluation and Cancelling Method to Restore Spatial Hearing. IEEE Access.

[12] Blauert J. (1996). Spatial Hearing: The Psychophysics of Human Sound Localization.

[13] Snapp H., Vogt K., Agterberg M.J.H. (2020). Bilateral bone conduction stimulation provides reliable binaural cues for localization. Hear. Res..

[14] Denk F., Ernst S.M.A., Ewert S.D., Kollmeier B. (2018). Adapting Hearing Devices to the Individual Ear Acoustics: Database and Target Response Correction Functions for Various Device Styles. Trends Hear..

[15] Denk F., Heeren J., Ewert S.D., Kollmeier B., Ernst S.M.A. Controlling the Head Position during Individual HRTF Measurements and Its Effect on Accuracy. Proceedings of the Fortschritte der Akustik—DAGA.

[16] Härmä A., Jakka J., Tikander M., Karjalainen M., Lokki T., Hiipakka J., Lorho G. (2004). Augmented Reality Audio for Mobile and Wearable Appliances. J. Audio Eng. Soc..

[17] Hoffmann P.F., Christensen F., Hammershøi D. Insert Earphone Calibration for Hear-Through Options. Proceedings of the AES 51st Conference on Loudspeakers and Headphones.

[18] Killion M.C. (1979). Design and Evaluation of High-Fidelity Hearing Aids. Ph.D. Thesis.

[19] Rämö J., Välimäki V. (2012). Digital Augmented Reality Audio Headset. J. Electr. Comput. Eng..

[20] Denk F., Schepker H., Doclo S., Kollmeier B. (2020). Acoustic Transparency in Hearables—Technical Evaluation. J. Audio Eng. Soc..

[21] Schlieper R., Preihs S., Peissig J. (2022). An Open Dataset of Measured HRTFs Perturbed by Headphones. Audio Engineering Society Convention 152.

[22] Schepker H., Denk F., Kollmeier B., Doclo S. (2020). Acoustic Transparency in Hearables—Perceptual Sound Quality Evaluations. J. Audio Eng. Soc..

[23] (2010). Acoustics-Audiometric Test Methods–Part 1: Pure-Tone Air and Bone Conduction Audiometry.

[24] Brainard D.H. (1997). The Psychophysics Toolbox Short Title: The Psychophysics Toolbox Corresponding Author. Spat. Vis..

[25] Pelli D.G. (1997). The VideoToolbox software for visual psychophysics: Transforming numbers into movies. Spat. Vis..

[26] Kleiner M., Brainard D., Pelli D., Ingling A., Murray R., Broussard C. (2007). What’s new in Psychtoolbox-3?. Perception.

[27] Farina A. (2000). Simultaneous Measurement of Impulse Response and Distortion with a Swept-Sine Technique. J. Audio Eng. Soc..

[28] Dietz M., Ewert S.D., Hohmann V. (2011). Auditory Model Based Direction Estimation of Concurrent Speakers from Binaural Signals. Speech Commun..

[29] Katz B.F.G., Noisternig M. (2014). A Comparative Study of Interaural Time Delay Estimation Methods. J. Acoust. Soc. Am..

[30] Tollin D.J., Yin T.C.T. (2009). Sound Localization: Neural Mechanisms. Encyclopedia of Neuroscience.

[31] Carpenter R.H., Reddi B.A., Anderson A.J. (2009). A simple two-stage model predicts response time distributions. J. Physiol..

[32] Carpenter R.H., Williams M.L.L. (1995). Neural computation of log likelihood in control of saccadic eye movements. Nature.

[33] Ausili S.A., Backus B., Agterberg M.J.H., van Opstal A.J., van Wanrooij M.M. (2019). Sound Localization in Real-Time Vocoded Cochlear-Implant Simulations with Normal-Hearing Listeners. Trends Hear..

[34] Ho J., Tumkaya T., Aryal S., Choi H., Claridge-Chang A. (2019). Moving beyond P Values: Data Analysis with Estimation Graphics. Nat. Methods.

[35] Van Wanrooij M.M., Bell A.H., Munoz D.P., Van Opstal A.J. (2009). The effect of spatial-temporal audiovisual disparities on saccades in a complex scene. Exp. Brain Res..

[36] Ohlenforst B., Zekveld A.A., Jansma E.P., Wang Y., Naylor G., Lorens A., Lunner T., Kramer S.E. (2017). Effects of hearing impairment and hearing aid amplification on listening effort: A systematic review. Ear Hear..

[37] Pichora-Fuller M.K., Kramer S.E., Eckert M.A., Edwards B., Hornsby B.W.Y., Humes L.E., Lemke U., Lunner T., Matthen M., Mackersie C.L. (2016). Hearing impairment and cognitive energy: The framework for understanding effortful listening (FUEL). Ear Hear..

[38] Best V., Kalluri S., McLachlan S., Valentine S., Edwards B., Carlile S. (2010). A Comparison of CIC and BTE Hearing Aids for Three-Dimensional Localization of Speech. Int. J. Audiol..

[39] Fletcher M.D., Zgheib J. (2020). Haptic Sound-Localisation for Use in Cochlear Implant and Hearing-Aid Users. Sci. Rep..

[40] Zheng Y., Swanson J., Koehnke J., Guan J. (2022). Sound Localization of Listeners With Normal Hearing, Impaired Hearing, Hearing Aids, Bone-Anchored Hearing Instruments, and Cochlear Implants: A Review. Am. J. Audiol..

